# COVID-19-related mobility reduction: heterogenous effects on sleep and physical activity rhythms

**DOI:** 10.1093/sleep/zsaa179

**Published:** 2020-09-11

**Authors:** Ju Lynn Ong, TeYang Lau, Stijn A A Massar, Zhi Ting Chong, Ben K L Ng, Daphne Koek, Wanting Zhao, B T Thomas Yeo, Karen Cheong, Michael W L Chee

**Affiliations:** 1 Centre for Sleep and Cognition, Yong Loo Lin School of Medicine, National University of Singapore, Singapore; 2 Health Promotion Board, Singapore; 3 Centre for Quantitative Medicine, Duke-NUS Medical School, Singapore; 4 Department of Electrical and Computer Engineering, National University of Singapore, Singapore; 5 N.1 Institute for Health, National University of Singapore, Singapore

**Keywords:** COVID-19, mobility restrictions, wearables, sleep, rest-activity rhythms, machine learning

## Abstract

**Study Objectives:**

Mobility restrictions imposed to suppress transmission of COVID-19 can alter physical activity (PA) and sleep patterns that are important for health and well-being. Characterization of response heterogeneity and their underlying associations may assist in stratifying the health impact of the pandemic.

**Methods:**

We obtained wearable data covering baseline, incremental mobility restriction, and lockdown periods from 1,824 city-dwelling, working adults aged 21–40 years, incorporating 206,381 nights of sleep and 334,038 days of PA. Distinct rest-activity rhythm (RAR) profiles were identified using k-means clustering, indicating participants’ temporal distribution of step counts over the day. Hierarchical clustering of the proportion of days spent in each of these RAR profiles revealed four groups who expressed different mixtures of RAR profiles before and during the lockdown.

**Results:**

Time in bed increased by 20 min during the lockdown without loss of sleep efficiency, while social jetlag measures decreased by 15 min. Resting heart rate declined by ~2 bpm. PA dropped an average of 42%. Four groups with different compositions of RAR profiles were found. Three were better able to maintain PA and weekday/weekend differentiation during lockdown. The least active group comprising ~51% of the sample, were younger and predominantly singles. Habitually less active already, this group showed the greatest reduction in PA during lockdown with little weekday/weekend differences.

**Conclusion:**

In the early aftermath of COVID-19 mobility restriction, PA appears to be more severely affected than sleep. RAR evaluation uncovered heterogeneity of responses to lockdown that could associate with different outcomes should the resolution of COVID-19 be protracted.

**Statement of Significance**

The COVID-19 pandemic has significantly impacted health and daily routines of many worldwide through widespread mobility restrictions. We analyzed longitudinal sleep/activity tracker data from ~1,800 office workers collected before the outbreak, through mobility restrictions, and culminating in lockdown. In addition to characterizing objective measures of sleep and physical activity (PA), we demonstrate how heterogenous groups are affected by using novel rest-activity rhythm and hierarchical clustering approaches. Contrary to popular expectation, sleep shifted later, duration increased, and social jetlag decreased. The substantial drop in PA is of greater concern. Adoption of our analytic methods may help identify groups at-risk in a protracted pandemic.

## Introduction

Adequate sleep and physical activity (PA) are two of the triad of lifestyle factors critical to multiple aspects of health and well-being [1–5]. Mobility restrictions imposed to contain the spread of COVID-19 (e.g. work from home, closure of social venues, and lockdown) have massively disrupted the daily routines of people worldwide [6]. This could lead to detrimental longer-term health consequences, particularly if lockdowns are protracted [4, 7].

During the pandemic, greater anxiety [8] and increased engagement on electronic media [9] for social engagement and news gathering could drive bedtimes later for some, curtailing sleep. On the other hand, for others, working from home [10] could afford greater flexibility in scheduling, save time from commuting, and reduce office-related stress, which could facilitate sleep health. Social jetlag (SJL) measures, which quantify the discrepancy between biological and social clocks [11] that often contribute to sleep loss, could in fact be reduced through greater flexibility in work hours. Recent survey-based studies [12, 13] appear to support improved sleep health but also point to instances where sleep quality may be adversely affected by greater sensitivity to stress [14].

PA benefits musculoskeletal, cognitive [15], cardiometabolic health [16], and sleep [17]. Additionally, exercise outdoors positively influences mental well-being [18] and morning light exposure serves to synchronize the circadian clock [19], restraining our innate tendency to sleep later over successive nights. Even if one does not have time for intentional exercise, walking on the way to and from work contributes significantly to PA [20, 21]. Mobility restrictions that limit commuting and social gatherings have resulted in a reduction of both structured and unstructured forms of PA [22, 23].

In addition to the duration of sleep and PA of themselves, their respective timing and distribution matter. For example, it may be preferable to accumulate PA across the day rather than to concentrate it within a short period but maintaining unhealthy levels of sedentary time [24]. How incremental mobility restriction upsets the rhythm of sleep and daytime PA is thus of broad public health interest.

In this study, we analyzed sleep and PA data from the “Health Insights Singapore” (hiSG) cohort. Since August 2018, over a thousand young working adults in Singapore were provided with a Fitbit Ionic wearable sleep and activity tracker to evaluate health behavior. This ongoing study provided us with a unique opportunity to characterize how COVID-19-associated mobility restrictions shifted sleep and PA patterns from previously established baselines using objective, longitudinal measurement in contrast to using surveys [9, 25]. We used machine learning to identify different rest-activity rhythm (RAR) profiles. This approach was also used to identify heterogenous sleep and PA transformations in different sociodemographic groups.

## Methods

### Data source

Data were obtained from the “Health Insights Singapore” (hiSG) study, a longitudinal population-health study by the Health Promotion Board using wrist-worn wearable technology. Initiated in August 2018, the study recruited 1,951 young adults working in the Central Business District aged 21–40 years. Participants were given devices (Fitbit Ionic, Fitbit Inc, San Francisco, CA) to track their activity/sleep and installed a mobile application to complete surveys over a period of 2 years. Participants were rewarded with points convertible to vouchers if they wore the tracker daily, logged sleep, meals, and completed surveys and were allowed to keep the device conditional upon meeting study requirements. Demographic, health, and lifestyle questionnaires were administered at study commencement. A second survey conducted in February 2020 was used to update any changes to family status, while a third survey was conducted in June 2020 to probe symptoms of depression using the revised Center for Epidemiologic Studies Depression Scale (CESD-R) [26]. To assess perceived sleep health during the lockdown, we extracted items from the CESD-R sleep subscale, which consisted of three questions. These questions asked about the number of days in the past 1–2 weeks an individual (1) perceived his sleep to be restless, (2) had trouble getting to sleep, and (3) slept much more than usual. The National Healthcare Group Domain Specific Review Board approved the study protocol. Informed consent was obtained from all subjects prior to study participation.

To evaluate the impact of the COVID-19 pandemic, we studied data gathered between January 2 and April 27, 2020, starting 3 weeks before the first case was reported in Singapore (“Baseline,” January 2–22) and ending 3 weeks into the lockdown enforced by the Singapore government (“Lockdown,” April 7–27). A 3-week period of increased restrictions (“Increased Restrictions,” March 17–April 6) was also included to assess the impact of incremental mobility restrictions before the lockdown. To properly evaluate shifts in habitual sleep and PA, we compared the 2020 data with an equivalent “Control” period in 2019; from January 3 to April 29, 2019. Only individuals who had valid data on *both* years after filtering (see below) were included. This comprised 206,381 nights of sleep and 334,038 days of PA from a final sample of 1,824 individuals. All dates presented refer to the morning of each sleep/daytime PA record, such that sleep always preceded PA for that date.

### Tracker-based data

Sleep and PA data for each participant were extracted from the Fitbit API. The PA data comprised daily total steps, moderate-to-vigorous physical activity (MVPA) minutes (sum of fairly and very active minutes), resting heart rate levels, and intraday step counts in 15-min intervals. Fitbit defines MVPA as activity of > 3METS (metabolic equivalents), and utilizes heart rate data to calculate active minutes for non-step-based activities such as weightlifting, yoga, and rowing [27]. For the comparison of measurements across time, data were filtered to remove days when participants did not wear the Fitbit for at least 8 h/day or when atypical activity levels were observed. This was defined as records with (1) total daily steps > 50,000, (2) total daily steps > 40,000 and sedentary minutes > 1,320 min, (3) sedentary minutes = 1,440 min, and (4) no resting heart rate and no steps measure. Between 1,041 and 1,562 (mean: 1,375) participants contributed to each timepoint for PA data. Wear time averaged 18–19 h (mean: 18.5 h) for each day. Not everyone contributed data on all the dates, but the large sample made it less likely that any individual’s data would significantly alter group means.

**Figure 1.**
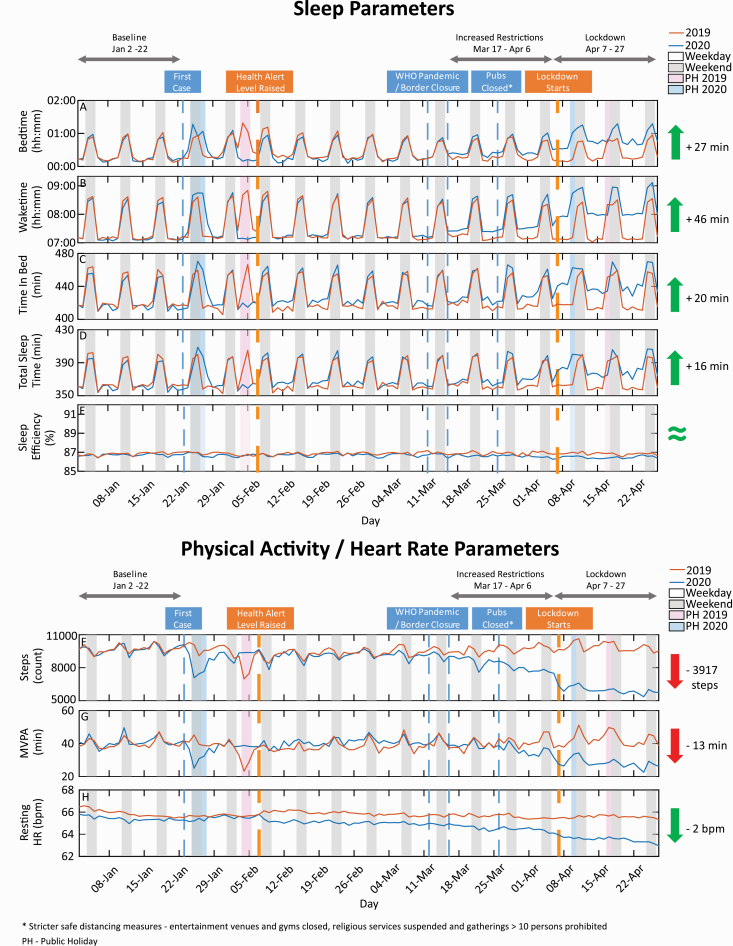
Time-series plots between January 2 and April 27, 2020 (blue curves) and January 3 and April 29, 2019 (red curves) for sleep (top panel) and PA/heart rate (bottom panel) parameters. (A) Bedtime, (B) Waketime, (C) TIB, (D) TST, (E) Sleep efficiency, (F) Step counts, (G) Time spent in MVPA, and (H) Resting heart rate. Weekends (gray-shaded regions) and public holidays (light blue- and pink-shaded regions) are also delineated. Dates reflect the “morning” of each record, such that sleep records always preceded PA. Dates in 2019 were shifted by 1 day in order to ensure a matching by day of the week. Key events during the COVID-19 pandemic period (“Baseline,” “Increased restrictions,” and “Lockdown”) are also indicated.

Sleep data consisted of bedtimes, wake times, time in bed (TIB), total sleep time (TST), and time spent awake after sleep onset (WASO). Sleep efficiency was computed as (100*TST/TIB) while SJL was defined as the difference between the midpoint of sleep on weekends and weekdays [11]. As in our prior work, we limited analyses to only nights with heart rate-derived sleep staged data, as this ensured proper wear time during the night, and excluded records manually adjusted by the user. Records that indicated <4 h TIB or >12 h TIB were also excluded from the calculation of sleep variables, as it could indicate possible split sleep sessions or inappropriate detection of sleep by the algorithm (e.g. long periods of sedentary activity after wake). In addition, to exclude atypical sleep periods, we removed sleep sessions that commenced between 08:00 am and 08:00 pm and split sleep sessions. Importantly, the number of records excluded did not materially differ for records pre-lockdown (4.61%) compared with during the lockdown (4.46%). After data filtering, between 766 and 1,063 (mean: 898) participants contributed to each timepoint for sleep data (Figure 1).

### Identification of RAR profiles

To integrate both amplitude and timing of PA and sleep periods across a 24 h period, we computed canonical RAR profiles for each day from each individual. To do this, 24 h intraday raw step counts were log-transformed using a natural log function. Each day consisted of 96 15-min interval bins, starting and ending at 12:00 am and 11:45 pm, respectively. All valid days (>13 h of total wear time) across the whole sample from January to April 2020 (125,851 days) were subsequently fed into a k-means clustering model to identify distinct clusters, or “basis sets” of daily RAR profiles. This approach enabled the quantitative characterization of RAR changes from baseline through lockdown.

The k-means++ algorithm in MATLAB R2016b (Mathworks, Inc., Natick, MA) was used to optimize initialization of the cluster centers [28]. This was done by random selection of the first cluster center, and subsequently choosing additional cluster centers from the remaining data points with probability proportional to their squared distance from the nearest existing cluster centers.

A range of cluster values was explored (*k* = 2–10) before settling on *k* = 4 for a parsimonious yet meaningful set of clusters. Euclidean distance was used as the distance metric.

### Identification of individual differences in changes to RAR profiles due to lockdown

To identify groups of individuals who showed similar changes in RAR profile composition with increasing mobility restrictions, we computed proportion of days spent in each RAR profile both pre- (January 1–April 6, 2020) and during (April 7–27, 2020) the lockdown for each individual. Agglomerative hierarchical clustering was performed using these proportions as feature values on participants with at least 60% of valid days pre- and during the lockdown (*N* = 670) using Ward’s method and the Euclidean distance metric. Missing days were excluded from the proportion calculation, such that proportion of time spent summed to 1 both pre- and during the lockdown. Supplementary analysis revealed that compared to excluded participants, included participants tended to be older (~1 year) and had a higher proportion of individuals married with children (27% vs. 19%).

The agglomerative hierarchical clustering method begins by considering each individual as a separate entity, and clustering individuals that are close together in distance. This process was repeated until all individuals were clustered. Inspection of the dendrogram helped identify a four-group solution.

### Statistical analysis

Paired *t*-tests were used to analyze differences between descriptive variables in the whole sample between specific periods of interest (i.e. “Incremental Restrictions,” “Lockdown,” and “Control”) and the “Baseline” period. For analyses of sociodemographic differences between the four RAR groups determined by hierarchical clustering, one-way analyses of variance (ANOVA) and Pearson’s chi-squared test of independence were used. In addition, 2 × 4 mixed ANOVAs were also performed on activity and sleep variables between the different groups with Time as the within-subject factor (“Baseline” and “Lockdown”) and Group as the between-subject factor. Only subjects who had a minimum of 5 weekdays and 2 weekends in each timepoint (“Baseline” and “Lockdown”) were included in this analysis (*N* = 667). For each variable, data points outside the 1.5 interquartile range were removed from analyses. All statistical analyses were performed in R version 3.6.1.

## Results

### Key characteristics of the sample and time-series data

Participants were between 21 and 40 years of age, 51.64% of whom were women. Most were office workers who commuted to Singapore’s Central Business District. They were relatively well educated (86.1% college degree) and earned a median salary of SGD 4,000–5,999. Singles comprised 57.8%, married persons with children 21.9%. Other details about sociodemographic features can be found in Table 1. The average weekday daily commute time reported in this sample was 1.92 h (*SD*: 0.71 h), so the time taken to travel to and from work could be estimated to be ~1 h each way.

**Table 1.** Sociodemographic characteristics of hiSG study participants

Characteristics	Statistics
Age in years, mean (*SD*), range	30.94 (4.62), 21–41
Gender, *N* (%)	
Female	941 (51.64)
Ethnicity, *N* (%)	
Chinese	1,718 (94.19)
Malay	31 (1.70)
Indian	48 (2.63)
Others	27 (1.48)
Family status, *N* (%)	
Single	934 (51.21)
Married with no children	376 (20.61)
Married with children	484 (26.54)
Separated/divorced/widowed	30 (1.64)
Household income in SGD, *N* (%)	
<$2k	180 (9.87)
$2k–$3.9k	721 (39.53)
$4k–$5.9k	511 (28.02)
$6k–$7.9k	172 (9.43)
$8k–$9.9k	96 (5.26)
≥$10k	144 (7.89)
Highest education, *N* (%)	
Degree	1,570 (86.07)
No degree	254 (13.93)

Figure 1 displays the average time courses of sleep (Figure 1, A–E) and activity (Figure 1, F–H) over a baseline period (January 2–22, 3 weeks prior to the first confirmed COVID-19 case in Singapore), to a period of incrementally increasing mobility restrictions (March 17–April 6), which eventually culminated in lockdown (April 7–27, first 3 weeks of lockdown; for a detailed timeline of events see Supplementary Figure S1; for a detailed breakdown of variables by phase see Tables 2–5). Data from 2020 are overlaid with data from the same period in 2019 for comparison.

**Table 2.** Sleep variables in 2020 by phase

	January 2–22, 2020 (Baseline)		March 17–April 6, 2020 (Increased restrictions)		April 7–27, 2020 (Lockdown)	
	Weekday (WD)	Weekend (WE)	Weekday (WD)	Weekend (WE)	Weekday (WD)	Weekend (WE)
Bedtime (hh:mm)	00:15 (01:08)	00:55 (01:23)	00:27 (01:12)***	00:58 (01:29)*	00:45 (01:20)***	01:16 (01:32)***
Waketime (hh:mm)	07:10 (01:09)	08:25 (01:31)	07:31 (01:12)***	08:38 (01:37)***	08:03 (01:18)***	09:00 (01:45)***
TIB (h)	6.92 (0.95)	7.49 (1.18)	7.07 (0.92)***	7.65 (1.16)***	7.28 (0.96)***	7.73 (1.18)***
TST (h)	5.99 (0.83)	6.50 (1.03)	6.11 (0.80)***	6.62 (1.03)***	6.30 (0.83)***	6.68 (1.03)***
WASO (h)	0.93 (0.21)	0.99 (0.25)	0.95 (0.21)*	1.03 (0.25)	0.99 (0.22)*	1.05 (0.26)
Sleep efficiency (%)	86.6 (2.4)	86.7 (2.5)	86.5 (2.3)*	86.6 (2.5)*	86.4 (2.26)***	86.5 (2.53)***
WE-WD TIB difference (min)	35 (76)		34 (70)		27 (69)***	
SJL (min)	58 (62)		50 (58)***		43 (57)***	

Values represent means and standard deviations for participants who provided data for each cell (*N* = 1,467–1,693). Pairwise comparisons are conducted with paired *t*-tests *on participants with complete data for both variables (N* = 1,330–1,552). Asterisks indicate significant differences from baseline (*p < 0.05, **p < 0.01, ***p < 0.001).

**Table 3.** PA variables in 2020 by phase

	January 2–22, 2020 (Baseline)		March 17–April 6, 2020 (Increased restrictions)		April 7–27, 2020 (Lockdown)	
	WD	WE	WD	WE	WD	WE
Steps (count)	9,344 (3,634)	8,992 (4,437)	7,796 (4,125)***	7,423 (4,679)***	5,284 (4,283)***	5,432 (4,692)***
Moderate-to-vigorous physical activity (min)	37.6 (27.9)	40.9 (37.5)	33.5 (29.9)***	36.0 (37.6)***	24.4 (28.2)***	27.2 (32.9)***
Resting heart rate (bpm)	65.7 (7.3)	65.8 (7.3)	65.0 (7.4)***	64.9 (7.4)***	64.2 (7.3)***	64.1 (7.37)***

Values represent means and standard deviations for participants who provided data for each cell (*N* = 1,654–1,806). Pairwise comparisons are conducted with paired *t*-tests *on participants with complete data for both variables (N* = 1,628–1,767). Asterisks indicate significant differences from baseline (*p < 0.05, **p < 0.01, ***p < 0.001).

**Table 4.** Control comparison of sleep variables in January 2019 and January 2020

	January 3–23, 2019		January 2–22, 2020	
	WD	WE	WD	WE
Bedtime (hh:mm)	00:13 (01:04)	00:56 (01:17)	00:15 (01:08)	00:55 (01:23)
Waketime (hh:mm)	07:10 (01:02)	08:35 (01:26)***	07:10 (01:09)	08:25 (01:31)
TIB (h)	6.93 (0.87)	7.65 (1.15)***	6.92 (0.95)	7.49 (1.18)
TST (h)	6.01 (0.75)	6.64 (1.01)***	5.99 (0.83)	6.50 (1.03)
WASO (h)	0.92 (0.21)**	1.00 (0.24)	0.93 (0.21)	0.99 (0.25)
Sleep efficiency (%)	86.8 (2.3)**	86.9 (2.4)*	86.6 (2.4)	86.7 (2.5)
WE-WD TIB difference (min)	42 (74)**		35 (76)	
SJL (min)	63 (60)**		58 (62)	

Values represent means and standard deviations for participants who provided data for each cell (*N* = 1,619–1,708). Pairwise comparisons are conducted with paired *t*-tests *on participants with complete data for both variables (N* = 1,468–1,610). Asterisks indicate significant differences from the January 2020 baseline period (*p < 0.05, **p < 0.01, ***p < 0.001).

**Table 5.** Control comparison of PA variables in January 2019 and January 2020

	January 3–23, 2019		January 2–22, 2020	
	WD	WE	WD	WE
Steps (count)	9,533 (3,144)**	9,200 (4,136)*	9,344 (3,634)	8,992 (4,437)
Moderate-to-vigorous physical activity (min)	37.5 (26.5)	41.5 (36.7)	37.6 (27.9)	40.9 (37.5)
Resting heart rate (bpm)	66.2 (7.5)***	66.3 (7.5)***	65.7 (7.3)	65.8 (7.3)

Values represent means and standard deviations for participants who provided data for each cell (*N* = 1,787–1,817). Pairwise comparisons are conducted with paired *t*-tests *on participants with complete data for both variables (N* = 1,768–1,800). Asterisks indicate significant differences from baseline (*p < 0.05, **p < 0.01, ***p < 0.001).

### Sleep changes with incremental mobility restrictions/lockdown

At baseline, the average weekday bedtime was 12:15 am, waketime was 07:10 am and total TIB was 6.92 h (TST: 5.99 h). Only 45% of the sample achieved the recommended sleep duration of 7 h (TIB) for this age group [29] on weeknights. This increased to 68% on weekends. On weekends, bedtime was 12:55 am, waketime was 08:25 am and total TIB was 7.49 h (TST: 6.50 h). The difference between weekend and weekday TIB was 35 min. SJL, an indicator of the mismatch between biological and social clocks was 58 min. Sleep efficiency, defined as the percentage of time spent actually asleep out of total TIB, and which is often considered as an objective marker of sleep quality, was generally maintained at 86.6% on weekdays and 86.7% on weekends.

During the period of incremental restrictions, sleep timing slightly but significantly shifted later compared to baseline (weekday: bedtime = 12:27 am, waketime = 07:31 am; weekend: bedtime = 12:58 am, waketime = 08:38 am, *p*’s < 0.05). While TIB and TST increased during this period (weekday: TIB = 7.07 h, TST = 6.11 h; weekend: TIB = 7.65 h, TST = 6.62 h, *p*’s < 0.001), sleep efficiency showed a significant but small decrease (weekday *SE* = 86.5%; weekend *SE* = 86.6%, *p*’s < 0.05). As for weekday–weekend differences, there was a reduction in SJL to 50 min.

Upon the imposition of lockdown, more drastic shifts in sleep in the same direction were observed. Average bed and wake times shifted by 21–53 min compared with baseline (weekday: bedtime = 12:45 am, waketime = 08:03 am; weekend: bedtime = 01:16 am, waketime = 09:00 am, *p*’s < 0.001), but as the delay in waketimes (weekday: +53 min, weekend: +35 min) was significantly larger than the delay in bedtimes (weekday: +30 min, weekend: +21 min; *p*’s < 0.001), this led to an overall increase in TIB and TST (weekday: TIB = 7.28 h, TST = 6.30 h; weekend: TIB = 7.73 h, TST = 6.68 h; *p*’s < 0.001) compared with baseline measures. About 64% of the sample achieved the recommended 7 h of sleep on weekdays during the lockdown—increasing by 19% from baseline measures, while 73% of the sample achieved this on weekends during the lockdown—increasing by 5% from baseline measures. Sleep efficiency was again slightly reduced compared to baseline (weekday: 86.4%; weekend: 86.5%, *p*’s < 0.001). Weekday–weekend differences, on the other hand, showed a significant reduction during lockdown (SJL = 43 min, weekend–weekday TIB difference = 27 min, *p*’s < 0.001).

To check on year-to-year stability in sleep measures, we contrasted the data from the January 2020 baseline to a comparable period in 2019 and found measures to be largely comparable (Tables 4 and 5).

### PA and resting heart rate changes with incremental mobility restrictions

PA measures showed a similar graded response to mobility restrictions and lockdown (Figure 1, F–H). At baseline, participants had an average daily step count around 9,344 steps on weekdays and 8,992 steps on weekends, and clocked between 38 (weekday) and 41 (weekend) min of MVPA. Resting heart rate levels hovered around 66 bpm at baseline (Figure 1, H).

In the period of incremental mobility restrictions, average step count reduced (weekday: 7,796 steps; weekend: 7,423 steps, *p*’s < 0.001), dropping further during lockdown (weekday: 5,284 steps; weekend: 5,432 steps, *p*’s < 0.001). MVPA similarly reduced during incremental restrictions (weekday: 34 min; weekend: 36 min, *p*’s < 0.001), and further in lockdown (weekday: 24 min; weekend: 27 min, *p*’s <0.001).

Resting heart rate also reduced during incremental restrictions (weekday: 65.0 bpm; weekend: 64.9 bpm, *p*’s < 0.001) and lockdown (weekday: 64.2 bpm, weekend: 64.1 bpm, *p*’s <0.001). Comparison of the baseline period in January 2020 to the same period in 2019 showed a few significant, but small differences (see Supplementary Table S1).

### Systematic patterns of heterogeneity in sleep and PA uncovered with RAR analysis

**Figure 2.**
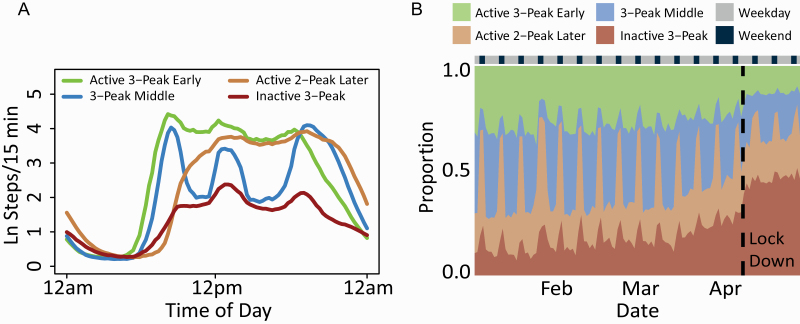
RAR profiles. (A) Centroids for the four key RAR profiles determined by k-means clustering of intraday step counts across 125,851 days from January to April 2020 from all participants for the “Active 3-Peak Early” (green curve, *n* = 28,723 days), “3-Peak Middle” (blue curve, *n* = 32,981 days), “Active 2-Peak Later” (brown curve, *n* = 30,635 days), and “Inactive 3-Peak” (red curve, *n* = 33,512 days) clusters. Clusters were differentiated by timing of morning rise and evening drop as well as magnitude of steps across the 24 h day. (B) Proportion of time spent in the four RAR profiles from January to April 2020. Pre-lockdown, weekday rhythms primarily consisted of the “Active 3-Peak Early” and “3-Peak Middle” profiles, while weekend rhythms mainly consisted of the “Active 2-Peak Later” and “Inactive 3-Peak” profiles. During the lockdown, a clear increase in the proportion of time spent in the “Active2-Peak Later” and “Inactive 3-Peak” profiles was observed, together with an attenuation of weekday–weekend rhythms.

In order to investigate concurrent changes to the magnitude of PA, as well as to the duration and timing of sleep and PA within an individual across days, we explored changes to 24 h RAR profiles with increasing mobility restrictions. We identified four distinct RAR profiles (Figure 2, A): “Active 3-Peak Early,” “3-Peak Middle,” “Active 2-Peak Later,” and “Inactive 3-Peak,” describing both magnitude as well as timing of preferred daytime activity. Pre-lockdown, weekdays tended to consist of more “Active 3-Peak Early” and “3-Peak Middle” days (Figure 2, B), indicating the strong influence of work as a frame around which life is organized (Peak 1: Traveling to work, Peak 2: Traveling to lunch, and Peak 3: Traveling home). In contrast, weekends tended to consist of more “Active 2-Peak Later” and “Inactive 3-Peak” days, indicating temporally less structured/lower magnitude of daytime activity. During the lockdown, both “weekend” patterns increased their expression during weekdays.

A control analyses performed on January 2019, January 2020 and during the lockdown separately revealed that before the restrictions were imposed, the clustered profiles were virtually identical (*r* > 0.99; Supplementary Figure S2, A–B). During the lockdown period, profiles became attenuated but were still highly similar to the profiles estimated using the full dataset (*r*: 0.84–0.98; Supplementary Figure S2, C).

### Individual differences in changes in RAR patterns due to lockdown

**Figure 3.**
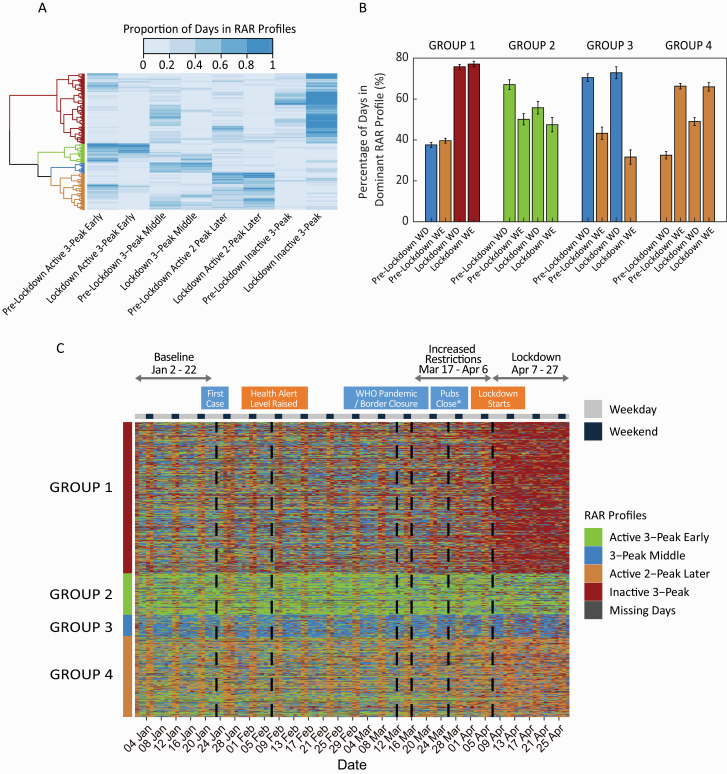
Identification of groups with similar RAR changes pre- and during the lockdown. (A) Hierarchical clustering of participants based on proportion of time spent in the four RAR profiles pre- and during the lockdown. Visual inspection of the dendrogram identified four groups of participants with similar changes in the patterns of RAR profiles pre- and during the lockdown. (B) Proportion of days spent in the dominant RAR profiles on weekdays and weekends for each group, pre- and during the lockdown. (C) RAR profiles across days (columns) by participant (rows) ordered by groups partitioned from the hierarchical clustering. Groups are colored by their dominant RAR profile color for ease of reference.

As a final analysis, we examined whether there were interindividual differences in how participants altered their rest-activity profiles. We calculated how often participants had shown each of the four identified RAR profiles (proportion of days spent in RAR profile) pre- and during the lockdown and used this to categorize participants into groups who showed similar changes to RAR profile composition pre-lockdown to during lockdown (using hierarchical clustering; see Figure 3, A).

**Figure 4.**
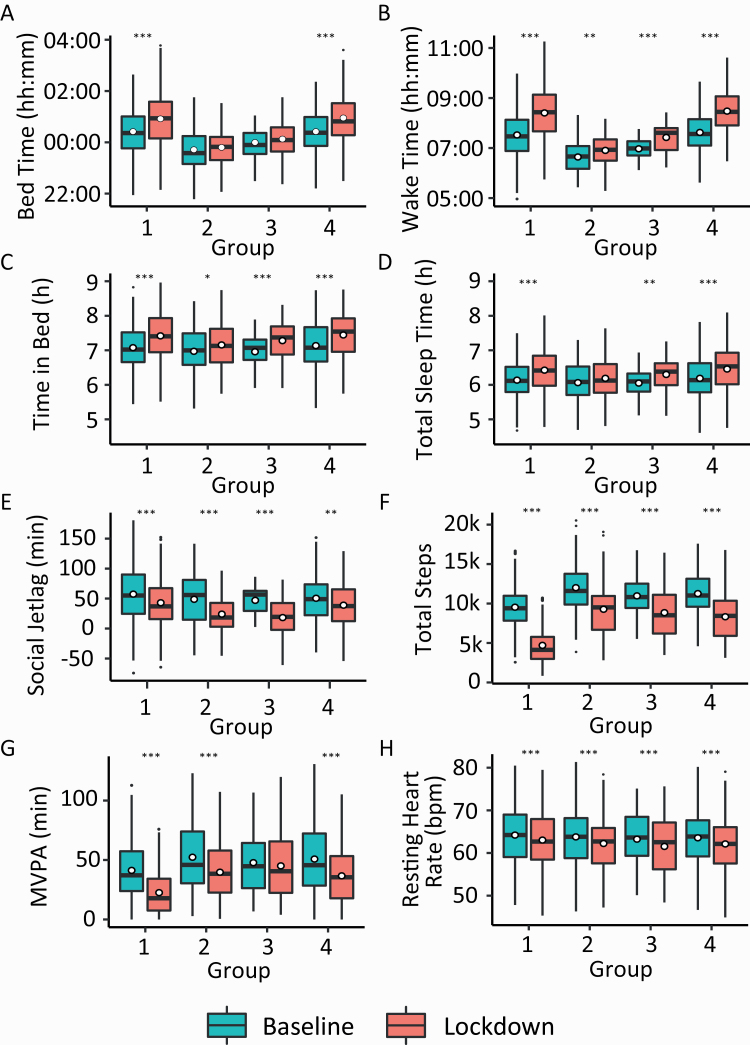
Boxplots for comparisons between groups identified by the hierarchical clustering, during the “Baseline” and “Lockdown” period. (A) Bedtime, (B) Waketime, (C) TIB, (D) TST, (E) SJL, (F) Step counts, (G) Time spent in MVPA, and (H) Resting heart rate. Asterisks denote significant pairwise comparisons between “Baseline” and “Lockdown” periods for each group. *p < 0.05, **p < 0.01, ***p < 0.001.

The largest group (Group 1: 51% of participants) predominantly showed the “3-Peak Middle” and “Active 2-Peak Later” profiles on pre-lockdown weekdays and weekends, respectively. During lockdown, they showed a large shift toward the “Inactive 3-Peak” RAR profile (Figure 3, B–C), and a clear abolition of weekday–weekend differences in RAR profiles. This group was the least physically active compared to the other three groups at baseline (9,517 steps in Group 1 compared with 12,011, 10,960, 11,275 steps in Groups 2, 3, and 4 respectively; all *p*’s < 0.001), and also showed the largest drop in step count—a 51% reduction in steps relative to baseline (−4,833 steps in Group 1 compared with −2,742, −2,098, and −2,966 in Groups 2, 3, and 4 respectively; all *p*’s < 0.001). This group also slept and woke later than Groups 2 and 3 (*p*’s < 0.05) (Figure 4, A–B, Table 6). TST was not adversely affected by the lockdown, and in fact, slightly increased by 17.5 min (*p* < 0.001). However, based on subjective reports of sleep quality, this group had difficulty falling asleep compared with Group 2 (*p* = 0.002), after controlling for age, sex, and family status. Interestingly, in contrast to their strongly attenuated weekday/weekend activity difference (baseline = 544 steps, lockdown = −338 steps, *p* < 0.001), there was only relatively, modest attenuation of weekday/weekend difference in TIB (baseline = −42 min, lockdown = −25 min, *p* < 0.001). This group was over-represented by younger, single persons.

**Table 6.** Sociodemographic, daily PA, and daily sleep measures by group

	Group 1 (M±*SD*) *n* = 270–344		Group 2 (M±*SD*) *n* = 74–94		Group 3 (M±*SD*) *n* = 37–50		Group 4 (M±*SD*) *n* = 148–182		*P*
Sociodemographics									
Age (y)	31.25 (4.46)^2,4^		32.87 (4.23)^1^		32.52 (4.05)		32.78 (4.23)^1^		<0.001
Sex-Females (%)	50.00		46.02		52.00		47.25		0.87
Ethnicity (%)									0.20
Chinese	94.48		93.62		98.00		91.76		
Malay	2.33		3.19		0.00		0.55		
Indian	2.04		1.06		2.00		3.85		
Others	1.16		2.13		0.00		3.85		
Education-degree holders (%)	84.88		84.04		100.00		83.52		0.03
Household income (%)									0.06
<$2k	10.17		11.70		2.00		10.99		
$2k–$3.9k	40.70		44.68		26.00		32.42		
$4k–$5.9k	26.16		13.83		34.00		28.57		
$6k–$7.9k	9.01		13.83		16.00		12.09		
$8k–$9.9k	6.11		9.57		6.00		6.60		
≥$10k	7.85		6.38		16.00		9.34		
Family status (%)									<0.001
Single	57.85^a^		35.11		40.00		35.17^b^		
Married with no children	20.64		7.45		28.00		18.68		
Married with children	20.06^b^		52.13^a^		30.00		43.96^a^		
Separated/divorced/widowed	1.45		5.32^b^		2.00		2.20		
Health									
BMI (kg/m^2^)	23.25 (4.12)		23.40 (3.88)		21.85 (2.25)^4^		23.61 (3.83)^3^		0.04
Sleep questionnaire†									
Restless sleep	1.12 (1.00)		0.91 (0.92)		0.97 (0.83)		0.89 (0.98)		0.11
Hypersomnia	0.69 (0.96)		0.54 (0.83)		0.59 (0.93)		0.57 (0.87)		0.74
Trouble falling asleep	0.91 (1.04)^2^		0.45 (0.72)^1^		0.57 (0.65)		0.68 (0.95)		0.001
	Baseline	Lockdown	Baseline	Lockdown	Baseline	Lockdown	Baseline	Lockdown	
Daily PA									
Total steps	9,516.81 (2,637.43)	4,683.34 (2,381.67)***	12,010.51 (3,055.46)	9,268.30 (3,374.22)***	10,959.78 (2,567.95)	8,861.49 (3,302.52)***	11,275.40 (2,780.22)	8,309.44 (2,934.34)***	<0.001 <0.001 <0.001
MVPA (min)	41.27 (24.25)	22.64 (18.33)***	52.22 (29.77)	39.78 (24.13)***	47.74 (25.96)	45.06 (28.47)	50.94 (29.04)	36.69 (23.16)***	<0.001 <0.001 <0.001
Resting heart rate (bpm)	64.24 (6.51)	63.04 (6.65)***	63.76 (6.51)	62.22 (6.37)***	63.28 (6.48)	61.57 (6.58)***	63.56 (6.23)	62.11 (6.49)***	0.38 <0.001 0.57
Daily sleep									
Bedtime (hh:mm)	00:25 (55.50)	00:55 (68.76)***	11:43 (46.68)	11:48 (44.50)	00:00 (37.00)	00:08 (40.79)	00:25 (50.61)	00:57 (57.62)***	<0.001 <0.001 <0.001
Wake time (hh:mm)	07:31 (54.38)	08:24 (64.52)***	06:39 (37.19)	06:54 (36.79)**	06:58 (25.46)	07:25 (32.71)***	07:38 (48.69)	08:28 (49.93)***	<0.001 <0.001 <0.001
TIB (h)	7.07 (38.64)	7.44 (43.05)***	6.97 (40.68)	7.16 (40.27)*	6.96 (30.25)	7.28 (34.91)***	7.15 (42.10)	7.48 (45.28)***	0.01 <0.001 0.11
TST (h)	6.13 (32.92)	6.43 (37.49) ***	6.05 (38.02)	6.19 (36.37)	6.05 (26.58)	6.30 (30.05) ***	6.19 (36.06)	6.46 (39.86) ***	0.03 <0.001 0.14
SJL (min)	57.63 (47.73)	43.08 (40.29)***	48.79 (43.09)	23.62 (30.05)***	47.42 (22.15)	17.97 (32.30)***	50.26 (40.75)	38.70 (34.73)**	<0.001 <0.001 0.04
WE-WD TIB difference (min)	41.82 (55.45)	24.63 (51.09)***	29.10 (46.50)	11.48 (38.66)*	31.27 (52.71)	17.41 (44.23)	41.64 (52.29)	24.62 (44.73)**	0.03 <0.001 0.99

†Values have been adjusted for age, sex, and family status.

^1^Significantly different from Group 1.

^2^Significantly different from Group 2.

^3^Significantly different from Group 3.

^4^Significantly different from Group 4.

^a^Cell’s observed proportion significantly higher than its expected proportion.

^b^Cell’s observed proportion significantly lower than its expected proportion.

*P* values under *P* column for daily PA, daily sleep, and SJL are ordered by main effect of group, main effect of time, interaction effect of group and time.

Significance values under daily PA, daily sleep, SJL, and weekend–weekday TIB difference refers to a significant difference from baseline after Bonferroni correction.

*p < 0.05.

**p < 0.01.

***p < 0.001.

Group 2 comprised 14% of persons and showed a dominant “Active 3-Peak Early” RAR profile (50%–70% of the time; Figure 3, B–C). This profile was dominant both pre-lockdown as well as during lockdown (although there was a slight reduction in proportion of the dominant profile on weekdays during the lockdown). This group was the most physically active, averaging around 12,011 steps before the pandemic and 9,268 steps when the lockdown was imposed. This group was over-represented by married couples with children, and tended to retain their habitual sleep/wake timings as well as TIB.

Group 3 expressed a clear weekday dominant “3-Peak Middle” RAR profile and weekend dominant “Active 2-Peak Later” RAR profile, which was preserved during the lockdown (Figure 3, B–C). This group had moderate step counts (between Groups 1 and 2) and moderate sleep–wake timings. Numerically, they also had the lowest drop in SJL measures during lockdown (−29 min), although this was only statistically significant compared to Group 1 (−15 min, *p* = 0.02) and Group 4 (−11 min, *p* = 0.01). Apart from a later sleep and wake timing, they bore significant similarities to Group 2 in terms of their consistency and intensity of PA. This group averaged 10,960 steps at baseline, dropped the least of all four groups and remarkably, maintained their habitual duration of MVPA even through lockdown (*p* = 0.50). This group was best able to maintain weekday–weekend differences in PA routines. Group 3 was strongly dominated by persons with a college degree but had even representation in terms of family status.

Group 4 was mainly characterized by a dominant “Active 2-Peak Later” RAR profile on both weekdays and weekends (Figure 3, B–C), which was only slightly attenuated during the lockdown as proportion of time spent in the dominant state increased on weekdays. It was the second largest group in the sample (27%) resembling Group 1 in terms of late sleep and wake timings (*p* = 0.99, *p* = 0.22, respectively) but its members were more physically active (*p* < 0.001), resembling Group 3 in consistency of steps, and intensity of overall PA (*p* = 0.48, *p* = 0.49, respectively). This group also had more individuals who were married with children.

## Discussion

Our data show that incremental mobility restrictions and lockdown to stem the spread of COVID-19 in Singapore had clear effects on sleep and PA patterns in young adult office workers. Across the entire sample, sleep timings shifted later, sleep duration increased, and weekend–weekday differences in sleep duration and timing (SJL) were substantially reduced. These findings align well with survey studies performed in other countries across the world (US, Europe, and China) [9, 12–14, 30]. Contrary to initial expectation, we found only a minimal reduction in sleep efficiency (indicative of sleep quality). Indices of PA (step count and MVPA), showed an expected sharp drop during mobility restrictions and lockdown, however resting heart rate (indicative of stress and cardiovascular risk), also significantly reduced in these periods.

Beyond providing objective confirmation and generalization of these overall changes found in other studies [9, 12–14, 30], the wearable data also allowed us to characterize interindividual differences in RAR profile compositions pre- and during the lockdown, based on temporal analysis of daily step counts.

### Sleep and PA under mobility restrictions restriction and lockdown

Contrary to the expectation that the pandemic would elevate anxiety which would be reflected in poorer sleep health [8, 25], we did not find support for this in our sample. Sleep efficiency, an objective marker of sleep quality was only minimally affected during the lockdown (86.5% vs 86.7% in baseline). Furthermore, although bedtimes were delayed, wake times were delayed even more, leading to total TIB *increasing* on both weekdays and weekends. The proportion of individuals who received at least 7 h of sleep on weekdays increased from 45% to 64%; on weekends this increased from 68% to 73%. The greater delays in bedtime and extension of sleep on weekdays compared to weekends also led to overall *decreased* SJL and weekend–weekday TIB difference during the lockdown. Time savings from commuting (mean total daily commute time ~110 min) could have freed up significantly more time to sleep. Travel time was second only to work time in having a reciprocal relationship to sleep in a major time-use study [31].

The overall pattern of results seems to reflect that the changes under mobility restrictions and lockdown might have actually benefited sleep. Apart from the increase in sleep duration, SJL measures are also reduced as individuals shift their daily schedules toward their biologically preferred sleep timings. As greater SJL/circadian misalignment has been associated with higher risk of depression [32], heart disease [33, 34], and adverse metabolic changes (e.g. type 2 diabetes and obesity) [35–37], these changes could in fact reflect better sleep habits during the lockdown. Although later sleep timings has also been shown to be associated with poorer health outcomes [34, 38] those observations may have been contributed by sleep curtailment from having waking up earlier than intended as a result of fixed morning work start times.

Changes in PA did follow the expected pattern of strong reductions under mobility restriction and lockdown [22, 23]. Overall, step count dropped from close to 10,000 steps a day at baseline, to nearly half that number during lockdown. MVPA followed a similar reduction. Such a drop in PA can be detrimental to health [5, 39] and mental well-being [40], and might be particularly worrisome when lockdowns are imposed for prolonged periods. An unexpected observation in this respect, the resting heart rate dropped by ~2 bpm. As resting heart rate is generally seen as an indicator of cardiovascular risk [41], we had expected this to be elevated over periods with reduced PA. The underlying cause for this reduction in resting heart rate (in the face of lesser activity), is not clear and it remains to be seen if this observation persists.

It might be slightly surprising to see the pattern of sleep and PA changes in this study, since a recent meta-analysis found a positive relationship between regular exercise and sleep [17]. The finding here that reduced PA and MVPA were associated with *gains* in TIB should not be taken as evidence to the contrary. Instead, this should serve as a reminder that associations between variables need to be interpreted with context in mind. Here, the highly unusual and disruptive effects of COVID-19 provide a vastly different environment than typical exercise/sleep association studies.

### RAR patterns show heterogenous changes due to lockdown

A particular advantage of the wearable-based activity data is that it allows tracking of PA (steps) over the course of a day. By analyzing the temporal distribution of these daily RAR, we were able to identify several distinct daily RAR profiles—some of which were more prevalent before lockdown, while others were more frequently observed during lockdown. Moreover, participants could be clustered into distinct groups that showed differential changes in RAR profile composition from pre-lockdown to lockdown. Some of these findings could perhaps be attributed to sociodemographic differences between these groups, the removal of social zeitgebers and reduction of morning light exposure from later waking.

Our RAR findings revealed that Group 1 could be at-risk of declining health in the long term, given the sharp drop in PA during lockdown to approximately half of that at baseline. Group 1 also evidenced poorer subjective sleep quality scores during the lockdown compared with Group 2, which could be driven in part by the larger drop in step counts given close links between levels of exercise and sleep quality [12]; however as this was only taken at one timepoint, we could not exclude the fact that this group suffered from poorer quality sleep in general. It is also concerning that these individuals comprised 51% of the sample which should merit targeted interventions if lockdowns continue. Further inspection of sociodemographic variables in these individuals revealed that they were predominantly single. Perhaps this group of individuals—with fewer childcare responsibilities and alternative social activity at home, work even longer hours resulting in less time for PA and a profound loss of differentiation between weekends and weekdays. Attenuation in weekday/weekend PA differences seen here could also reflect social isolation [42] that has long-term consequences on mental well-being. This group of individuals could benefit from setting regular routines to provide time for rest, PA and work, and to sleep and wake at fixed times at a time when social cues are disrupted.

By contrast, Group 2 who woke up the earliest and also slept earlier are over-represented by persons who are married with children. Having the responsibility to care for the latter would serve to drive more temporally structured activity, leading to the preservation of a three-peaked RAR even during the lockdown. This group tended to better retain their habitual sleep/wake timings as well as TST.

Group 3, who had relatively better sleep and PA habits during the lockdown—fairly consistent bedtimes and waketimes, large reductions in SJL, preserved MVPA and maintenance of ~10,000 daily step counts, consisted entirely of college degree holders (100%), which could have made them better informed about adverse effects of unhealthy behaviors in the long-term. Greater sleep regularity [43–45] and lesser weekend–weekday differences [39] in sleep timing are considered favorable to health. Although not statistically significant, there was also a trend for resting heart rate, a proxy for cardiovascular risk, to diverge between Group 1 and Group 3.

In industrialized societies, social zeitgebers play an important role on sleep schedules [46]. For persons in Groups 1 and 4, not having to wake up early to go to the office, likely surfaced innate delayed sleep timing, thus lessening the impact of keeping to traditional work hours on sleep schedules. The overall later timings of bedtime and waketime, even for earlier sleepers and risers relative to results published elsewhere [47], could also be indicative of Singapore’s “westerly” position relative to its assigned time zone. Later bedtimes and shorter nocturnal sleep co-occur with chronic, longitude-related exposure to later evening light [48, 49]. Delayed sleep schedules could also be further exacerbated by reduced morning and afternoon exercise, as these tend to advance the circadian clock [50].

Finally, the robustness of RAR profiles revealed over comparable weeks in January 2019 and 2020 speaks to their utility as basis patterns whose automated detection reduces the dimensionality of longitudinal sleep and PA data by integrating both measures of sleep timing and distribution of PA across a 24 h day. This could simplify identification of persons who might benefit from customized counsel during extended lockdowns. When data on the long-term impact on health, well-being or productivity measurements emerge, RAR may be even refined as a public health predictive tool, especially when combined with smartphone apps [51].

### Strengths and limitations

The data reported here were collected as part of an ongoing population-health study. In the context of understanding COVID-19 related changes, this approach has several clear strengths and limitations. First of all, having access to wearable-based sleep and PA records for over 1,800 participants provides a particular opportunity to assess the time courses of change due to the COVID-related restrictions and lockdown. This complements existing survey studies [9, 12–14, 30] with objectively measured data and can aid to further generalize findings. A clear advantage is that the data was collected over the full period spanning from a pre-pandemic baseline, to increasing mobility restrictions, to lockdown, which allowed for clear comparisons across these distinct time periods. Moreover, data for an equivalent period in 2019 were available, providing an additional reference against which the 2020 pandemic situation could be contrasted against. Importantly, the data reported were collected from the same participants whose data was available over the 2 years. This is an important distinction from “trend data” gathered from public convenience-sample sites like Google Trends, or reports from wearable manufacturers, which do not provide such direct within-individual comparison. As Singapore lies next to the equator, our sleep timing data is not confounded by seasonal variations [41]. Another advantage of the wearable-based methodology was that activity profiles could be tracked with a high level of granularity. This allowed us to examine sleep/activity profiles in much finer detail than just the daily averages, and to identify significant heterogeneity in RAR profiles and their changes due to lockdown.

There may be some concerns about the reliability of consumer-grade sleep tracking. However, validation studies on Fitbit devices conducted by several research groups (see [52] for a review of validation studies for sleep, and [53] for PA) have been encouraging, demonstrating overall high correlation values (*r*’s > 0.70) compared to gold standard polysomnography/research-grade accelerometers. We have also previously shown the overall fidelity of multiday sleep tracking in parallel with PSG [54]. The Fitbit is not configured to capture short daytime naps, which could increase in frequency when individuals work from home. These potential limitations notwithstanding, the additional insights provided by objectively measured sleep and activity recordings at this scale are highly valuable. It can be expected that the use of commercial sleep/activity trackers will be increasingly prevalent in future research.

The interpretation of sleep changes might be served by additional information about sleep disorders and/or chronotype, and more detailed information about occupation work schedules may add insights to the heterogeneity analysis. Interpretation of the lowered resting heart rate findings could also have benefited from information about participants’ self-perceived stress. As most of the participants have existing jobs, which for the present time appear to be preserved by various governmental financial supports, the sleep patterns here are likely, less affected than for a socioeconomically distressed sample. Finally, as further lockdowns are likely and the period of exposure to widespread mobility restrictions may be extended, the findings herein may be time-bounded in their generalizability.

## Conclusion

Longitudinal monitoring of sleep and PA patterns through incremental mobility restrictions culminating in lockdown to stem the spread of COVID-19 in Singapore showed that adult office workers evidenced delays to sleep timings, increases to sleep duration, decreases to weekend–weekday differences in sleep timing and duration and reductions to step counts, MVPA, and resting heart rate levels. However, heterogenous changes between groups of individuals could be observed upon inspection of RAR profiles pre- and during the lockdown. Widescale adoption of the methods described here may help identify persons or groups at-risk should the pandemic be protracted.

## Supplementary Material

zsaa179_suppl_Supplementary_Figures

## Data Availability

This article appears as a preprint at https://arxiv.org/abs/2006.02100
