# Supplementary material for: COVID-19-related mobility reduction: heterogenous effects on sleep and physical activity rhythms
Source: Sleep. 2020 Sep 11;44(2):zsaa179. doi: 10.1093/sleep/zsaa179 (PMC7543649; doi:10.1093/sleep/zsaa179)
Supplement: zsaa179_suppl_Supplementary_Figures [file zsaa179_suppl_supplementary_figures.docx]

**COVID-19 Related Mobility Reduction: Heterogenous Effects on Sleep and Physical Activity Rhythms**

Ju Lynn Ong^1^, TeYang Lau^1^, Stijn A.A. Massar^1^, Zhi Ting Chong^2^, Ben K.L. Ng^2^, Daphne Koek^2^, Wanting Zhao^2,3^, B.T. Thomas Yeo^1,4,5^, Karen Cheong^2^ and Michael W.L. Chee^1^*

^1^Centre for Sleep and Cognition, Yong Loo Lin School of Medicine, National University of Singapore, 12 Science Drive 2, Singapore 117549, Singapore.

^2^Health Promotion Board, 3 Second Hospital Ave, Singapore 168937, Singapore.

^3^Centre for Quantitative Medicine, Duke-NUS Medical School, 2 College Rd, Singapore 169857, Singapore.

^4^Department of Electrical and Computer Engineering, 4 Engineering Drive 3, National University of Singapore, Singapore 117583, Singapore.

^5^N.1 Institute for Health, National University of Singapore, 28 Medical Drive, Singapore 117456, Singapore.

* Corresponding author:

Michael W.L. Chee

Professor and Director,

Center for Sleep and Cognition,

Yong Loo Lin School of Medicine,

12 Science Drive 2,

National University of Singapore,

Singapore 117549.

Email: [michael.chee@nus.edu.sg](mailto:michael.chee@nus.edu.sg)


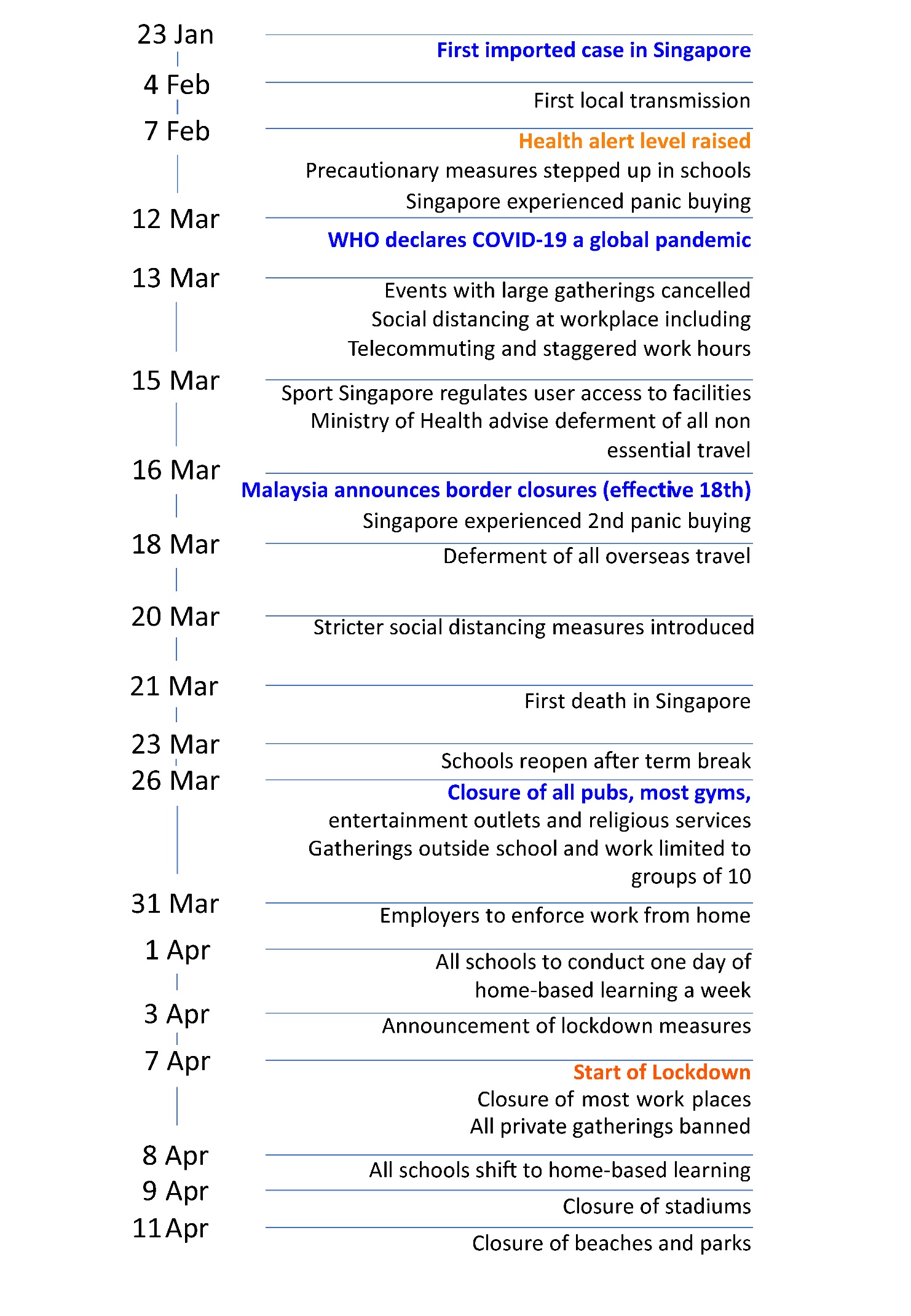
**Figure S1.** Timeline of key COVID events in Singapore.

**
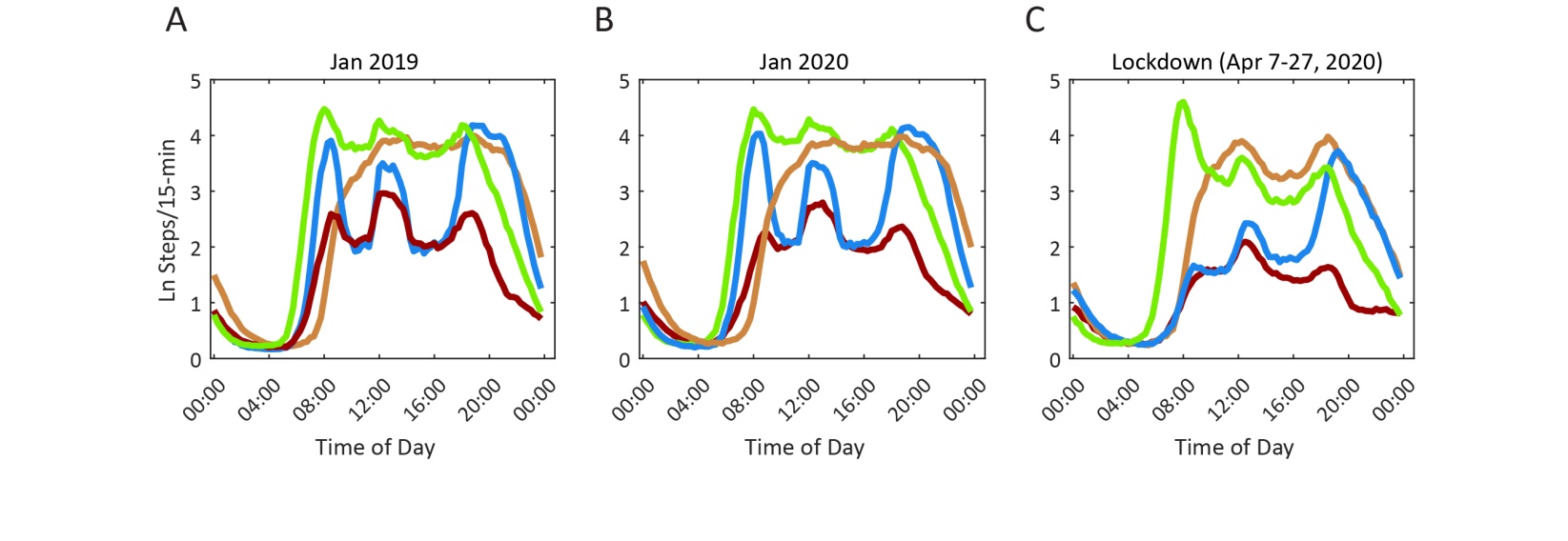
**

**Figure S2.** k-means clustering of RAR profiles in Jan 2019, Jan 2020 and during the lockdown. RAR profiles were highly similar between Jan 2019 and Jan 2020 (r > 0.99). After the lockdown RAR profiles were slightly attenuated, but still highly similar to the profiles estimated using the full dataset from Jan-Apr 2020 (r: 0.84-0.98).

**
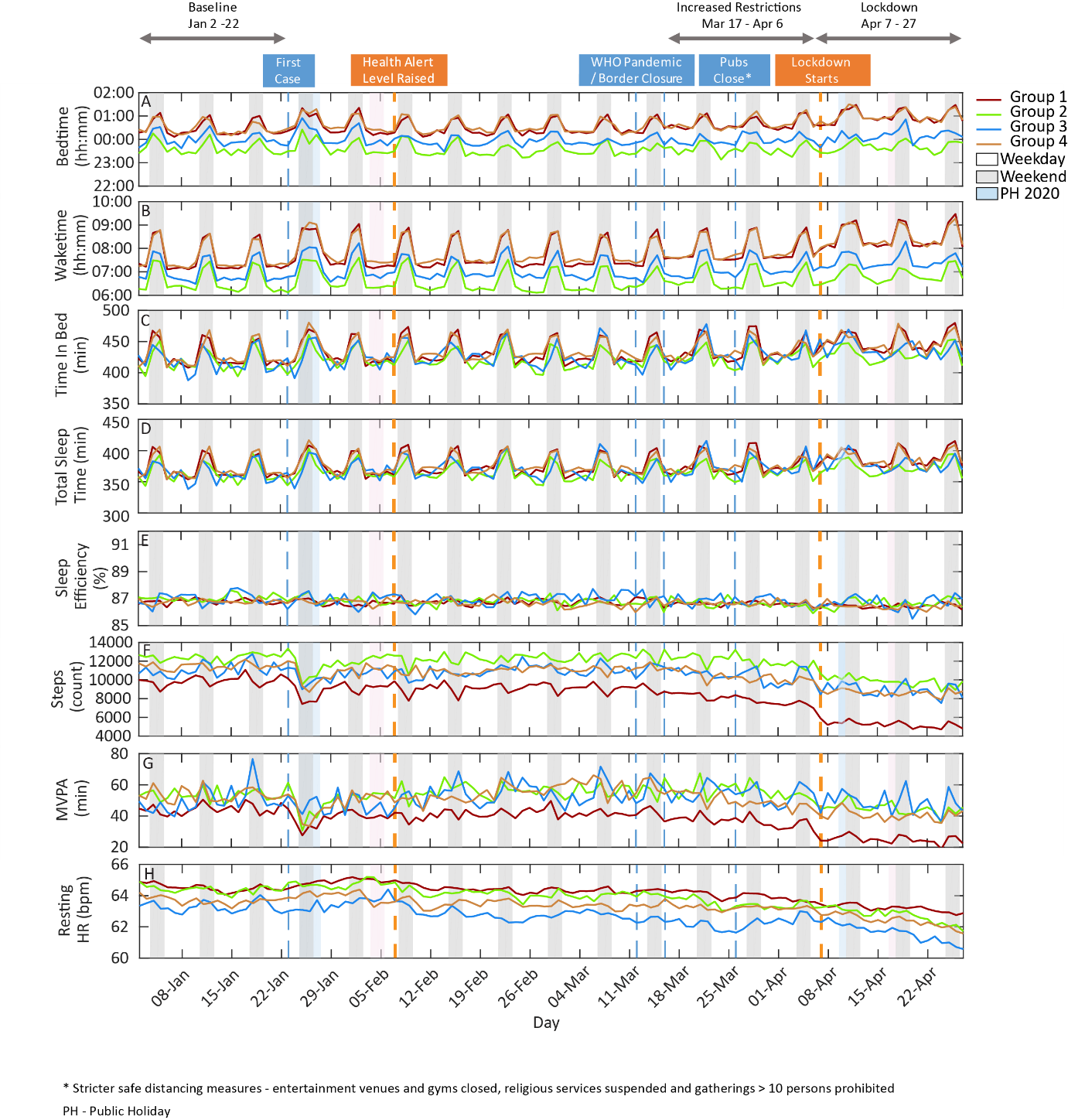
**

**Figure S3.** Time series plots between Jan 2 - Apr 27, 2020 (blue curves) and Jan 3 - Apr 29, 2019 (red curves). (A) Bedtime, (B) Waketime, (C) Time In Bed, (D) Total Sleep Time, (E) Sleep Efficiency, (F) Step Counts, (G) Time spent in Moderate-to-Vigorous Physical Activity (MVPA) and (H) Resting Heart Rate for the 4 groups identified by the hierarchical clustering. Weekends (gray shaded regions) and public holidays (light blue and pink shaded regions) are also delineated. Dates reflect the ‘morning’ of each record, such that sleep records always preceded physical activity. Key events during the COVID-19 pandemic period (‘Baseline’, ‘Increased Restrictions’, and ‘Lockdown’) are also indicated.
